# Release dynamics of nanodiamonds created by laser-driven shock-compression of polyethylene terephthalate

**DOI:** 10.1038/s41598-024-62367-7

**Published:** 2024-05-28

**Authors:** Ben Heuser, Armin Bergermann, Michael G. Stevenson, Divyanshu Ranjan, Zhiyu He, Julian Lütgert, Samuel Schumacher, Mandy Bethkenhagen, Adrien Descamps, Eric Galtier, Arianna E. Gleason, Dimitri Khaghani, Griffin D. Glenn, Eric F. Cunningham, Siegfried H. Glenzer, Nicholas J. Hartley, Jean-Alexis Hernandez, Oliver S. Humphries, Kento Katagiri, Hae Ja Lee, Emma E. McBride, Kohei Miyanishi, Bob Nagler, Benjamin Ofori-Okai, Norimasa Ozaki, Silvia Pandolfi, Chongbing Qu, Philipp Thomas May, Ronald Redmer, Christopher Schoenwaelder, Keiichi Sueda, Toshinori Yabuuchi, Makina Yabashi, Bratislav Lukic, Alexander Rack, Lisa M. V. Zinta, Tommaso Vinci, Alessandra Benuzzi-Mounaix, Alessandra Ravasio, Dominik Kraus

**Affiliations:** 1 Institut für Physik, Universität Rostock, Albert-Einstein-Str. 23-24, 18059 Rostock, Germany; 2 Helmholtz-Zentrum Dresden-Rossendorf, Institute of Radiation Physics, Dresden, 01328 Germany; 3 China Academy of Engineering Physics, Shanghai Institute of Laser Plasma, Shanghai, 201800 China; 4 LULI, CNRS, CEA, Ecole Polytechnique-Institut Polytechnique de Paris, Sorbonne Université, Palaiseau, 91128 France; 5 School of Mathematics and Physics, Queen’s University Belfast, Belfast, Northern Ireland BT7 1NN UK; 6 SLAC National Accelerator Laboratory, Menlo Park, CA 94025 USA; 7 Stanford University, Stanford, CA 94305 USA; 8 European Synchrotron Radiation Facility, 71 avenue des Martyrs, CS 40220, 38043 Grenoble, France; 9 The Centre for Earth Evolution and Dynamics (CEED), University of Oslo, Oslo, 0371 Norway; 10 European XFEL, Schenefeld, 22869 Germany; 11 Materials Science and Engineering, Stanford University, Stanford, CA 94305 USA; 12 RIKEN SPring-8 Center, Hyogo, 679-5148 Japan; 13 Graduate School of Engineering, Osaka University, Suita, Osaka 565-0871 Japan; 14 Photon Pioneers Center, Osaka University, Suita, Osaka 565-0087 Japan; 15 Sorbonne Université, Institut de Minéralogie, de Physique des Matériaux et de Cosmochimie, IMPMC, Muséum National d’Histoire Naturelle, UMR CNRS 7590, 75005 Paris, France; 16 Japan Synchrotron Radiation Research Institute (JASRI), Hyogo, 679-5198 Japan

**Keywords:** Nanoparticles, Synthesis and processing, Laboratory astrophysics, Inner planets

## Abstract

Laser-driven dynamic compression experiments of plastic materials have found surprisingly fast formation of nanodiamonds (ND) via X-ray probing. This mechanism is relevant for planetary models, but could also open efficient synthesis routes for tailored NDs. We investigate the release mechanics of compressed NDs by molecular dynamics simulation of the isotropic expansion of finite size diamond from different *P*-*T* states. Analysing the structural integrity along different release paths via molecular dynamic simulations, we found substantial disintegration rates upon shock release, increasing with the on-Hugnoiot shock temperature. We also find that recrystallization can occur after the expansion and hence during the release, depending on subsequent cooling mechanisms. Our study suggests higher ND recovery rates from off-Hugoniot states, e.g., via double-shocks, due to faster cooling. Laser-driven shock compression experiments of polyethylene terephthalate (PET) samples with in situ X-ray probing at the simulated conditions found diamond signal that persists up to 11 ns after breakout. In the diffraction pattern, we observed peak shifts, which we attribute to thermal expansion of the NDs and thus a total release of pressure, which indicates the stability of the released NDs.

## Introduction

For many decades, high-pressure, high-temperature states of materials have been investigated in the laboratory, mainly pursuing fundamental science, materials science, and planetary physics^1–7^. One of the established experimental approaches, laser-driven dynamic compression, has made significant advances in recent years due to the advent of bright X-ray sources, such as X-ray free electron lasers and the newest generation of synchrotron light sources^8–10^. These allow for precise *in situ* measurements of material response, structural transitions, and pressure-induced chemistry, which have started to revolutionise the microscopic understanding of matter dynamically compressed to pressures in the regime of several million atmospheres and temperatures of several thousand Kelvins^11^. The properties of materials during unloading are equally important for understanding the formation and stability of phases. The low-temperature end of the so-called warm dense matter regime is poorly understood and thus, modelling structural transitions and chemistry is extraordinarily difficult and experimental benchmarks are vital^12,13^. Recent dynamic compression experiments of plastic materials combined with in situ X-ray scattering have found a surprisingly fast phase separation of carbon and hydrogen and the formation of nanodiamonds (NDs) on a sub-nanosecond timescale^4,14–16^. In previous experiments, nucleation rates of NDs in laser-driven shocks were higher than $10^{29}$ ${{\hbox {m}}^{-3}\hbox {s}^{-1}}$^4^. This finding may open a new route for an efficient synthesis of tailored NDs, e.g., with well-controlled size and specific color centres, besides implications for planetary interiors.

NDs, mostly synthesized by detonating high explosives, have been commercially available since the 1990s and are used in various applications from industry to catalysis^17–20^. However, the controlled formation of NDs of just a few nanometers in size and including specific color centers directly in the formation process remains difficult^21^. Moreover, surface chemistry is essential for catalytic reactions and strongly depends on the synthesis method and finishing^22^. The amination, the attachment of amine groups to the ND, enables the binding of bioactive molecules to the NDs surface. Detonation NDs are well-suited for additives in lubricants, while their hydrogenation raises the efficacy of radiation therapy for cancer cells^22–24^. Therefore, studying the surface chemistry of ND produced by shock compression is of great interest. Moreover, investigating the dynamic formation of NDs from laser-shocked plastics will improve the understanding of detonation synthesis. Intact recovery of ND from laser-shocked plastics remains elusive. This motivates detailed studies of their release behaviour. Understanding the stability of NDs on different shock release paths might help in tailoring the shock conditions and possible tamper windows for intact recovery. The ND dynamics at high ejecta and impact velocities are also relevant for designing possible ND catchers. Furthermore, knowing the exact phase volume and atomic ratios within the sample where diamonds nucleate is valuable information.

This study analyses ND formation and subsequent release dynamics from shock-compressed polyethylene terephthalate (PET) via in situ X-ray diffraction (XRD) in two separate experiments. A Velocity Interferometer System for Any Reflector (VISAR) determined the Hugoniot state, which can be related to temperature via Equation Of State (EOS) data. The free surface velocity was compared to data obtained from a high-speed camera in transversal geometry. To understand quenching of NDs from high pressures to ambient conditions after shock breakout, we performed Molecular Dynamics (MD) simulations at different *P*-*T* conditions. To simplify our system, our simulations neglect any influence of surrounding plastic material, and instead study the ND’s stability itself. This isolated view might introduce systematic errors, and indeed we observe notable discrepancies between simulation and experiment. However, qualitative trends might still give valuable insight into the bigger picture of recovery experiments. These simulations were performed in three different steps after the initial equilibration phase. First the expansion of the NDs was simulated to zero pressure^25,26^. Afterwards, the expanded system was cooled to room temperature. Then another equilibration at ambient conditions was performed, assessing long-term stability. We analysed results of the structural integrity of NDs and their thermodynamic properties along the release path. The high temporal and spatial resolution of the MD results along the release path from different *P*-*T* states helps in understanding ND stability upon quenching. Because MD simulations are dependent on the used potentials, and predicting absolute values requires a very good description of the underlying physics at all points during the simulation, we are using these simulations as a qualitative guide for trends in the release process. The simulations agree qualitatively with the experimental observations, giving direct implications for future experiments.

## Results

### Expansion simulation

In the simulation, an ND cube with $8 \times 8 \times 8$ cells and a total of 4096 atoms was initialized at different states on the PET Hugoniot curve, which defines all points in *P*-*T* space reachable by a single shock in a particular material. The chosen equilibration temperatures were 2000 K, 3000 K, 4000 K, and 5000 K at the corresponding pressure along the PET Hugoniot^27^.

**Figure 1:**
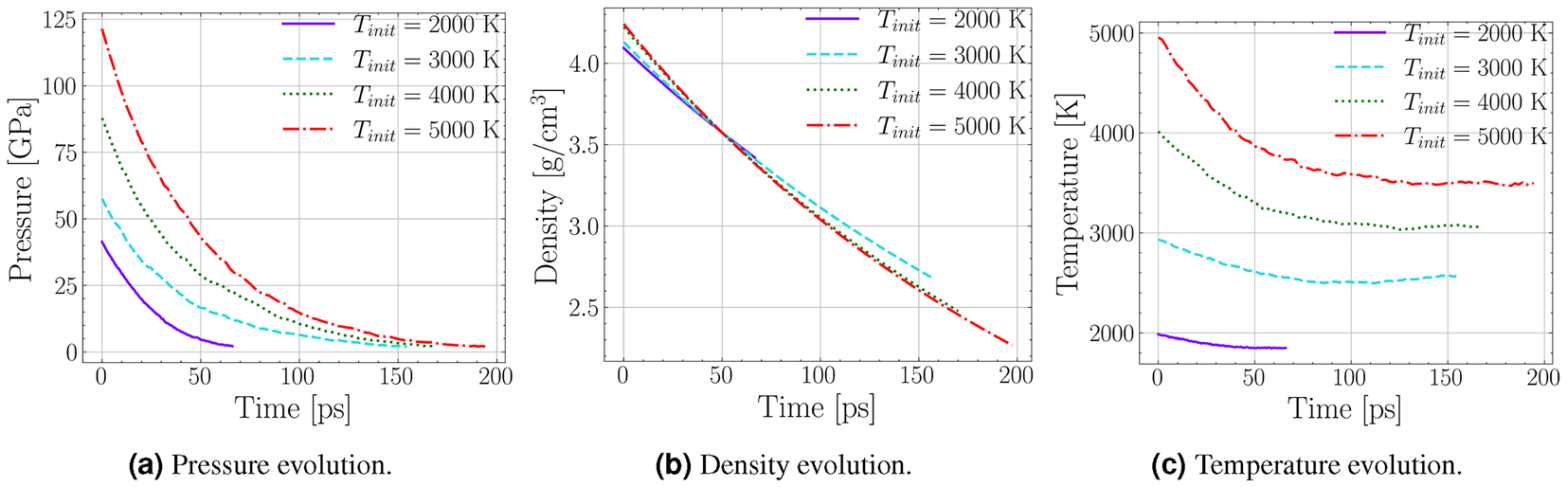
Evolution of the thermodynamic properties in the expansion phase of an expanding ND cube. Each line represents a different initial point on the principal Hugoniot where a ND cube was equilibrated. **(a)** The simulation is halted when the final pressure drops to zero. This takes longer for an initial higher pressure. The maximal drop rates occur for the highest pressure. **(b)** The decrease in density and the final densities are very similar except for the 2000 K case. Here, the final density is close to ambient diamond. **(c)** The final temperature increases with the initial state. The maximal cooling rates are observed right at the beginning and increase with initial temperature.

After the equilibration at the Hugoniot states, we expanded the lattice using a constant dilation rate. For a detailed description of the simulation, see Section Methods: Molecular Dynamics Simulation. As the diamond expands and the pressure is reduced, the temperature decreases simultaneously. The pressure reduces continuously until it drops below zero, and the simulation is halted. Figure 1a shows the release from different initial temperatures and pressures. The total time it takes the system to release to zero pressure depends on the initial state, given a constant dilation rate. At the highest initial Hugoniot state at $T=5000$ K and $P=122$ GPa, the simulation takes 197 ps to release to zero pressure. The corresponding densities are displayed in Fig. 1b. The release curves follow the same functional shape, but the final density values differ substantially. In the simulation starting at 2000 K the pressure reaches zero just below the ambient diamond density at 3.5 $\hbox {g cm}^{-3}$. This is because the entire diamond cube stays intact with only minor surface modifications, so the final density will be close to an expanded diamond cube. In the simulations where less diamond remains intact, the density is approaching the value of ambient graphite 2.3 $\hbox {g cm}^{-3}$.

**Figure 2:**
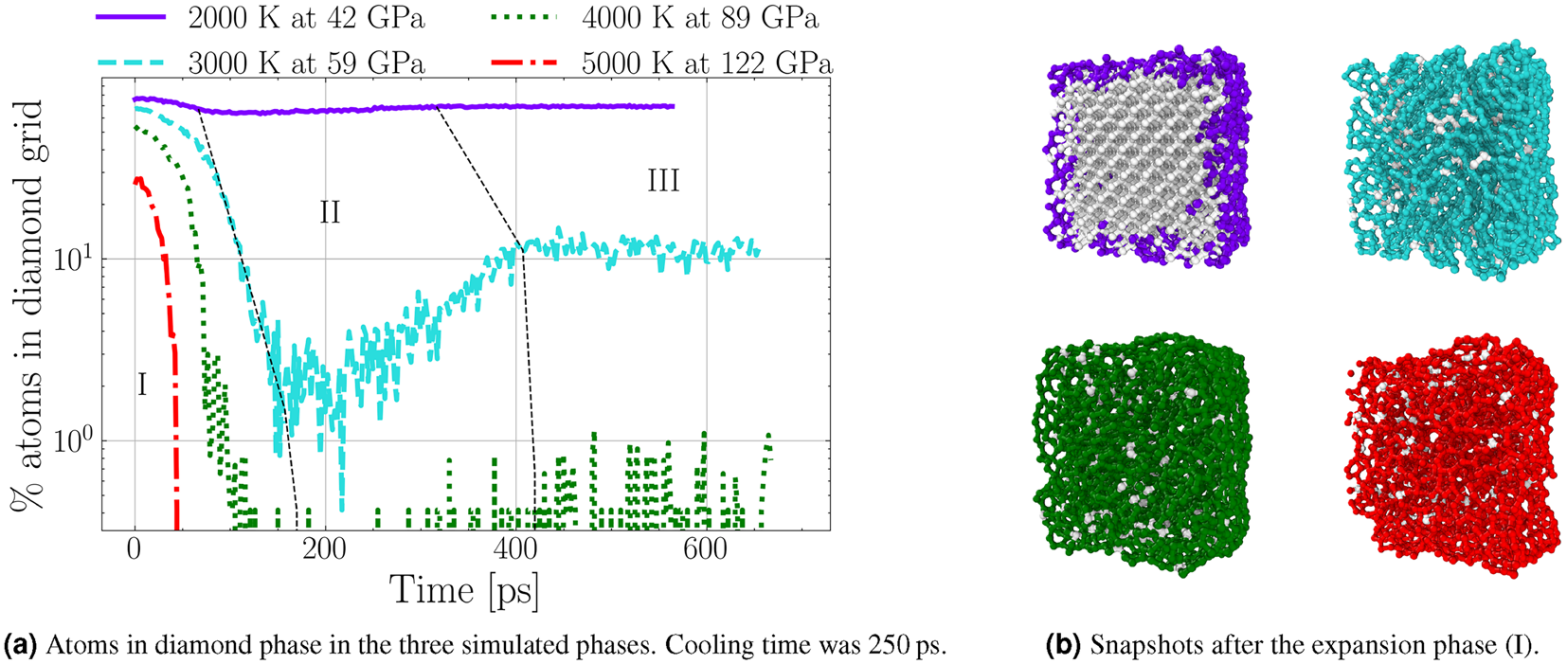
**(a)** Diamond atoms during the entire simulation. Dotted black lines separate the three simulation phases: (I) expansion, (II) cooling, and (III) equilibration. Each coloured line represents one initial Hugoniot state. The disintegration of the diamond lattice in the expansion and partial recrystallisation is clearly visible. **(b)** Nearest neighbour bonds of the NDs after the release to zero pressure. The coloured atoms are $sp^{2}$ bonded and the light atoms are $sp^{3}$ bonded. The hexagonal bond structure which resembles fullerene-like shells is clearly visible in $T_{init}= 3000$  K and $T_{init}= 4000$  K simulations.

As the pressure drops and the volume increases, the system does volume work by the expansion against the surrounding high pressure environment and the temperature is reduced. The temperature as a function of time is displayed in Fig. 1c. The rate of temperature decrease is highest at the beginning, and almost vanishes at the end. In the 2000 K simulation, the temperature drop is about $\Delta T = 140$ K, while for the highest temperature of 5000 K, it is about $\Delta T=1500$ K. The higher the initial pressure, the higher the volume work that is done against this pressure by the system, and the higher the drop in temperature. Over all simulated states, this corresponds to average cooling rates from $1.8\times 10^{12}$  to $7.6\times 10^{12}$ $\hbox {K}/\hbox {s}$, which are comparable to MD simulations that were carried out on nanoparticles^28^. While pressure and temperature are dropping, the diamond lattice can become unstable, and defects can start from the diamond surface and grow inwards^29^. The non-diamond carbon atoms are forming chain like molecules with limited mobility. Figure 2a shows the percentage of atoms in a diamond lattice over the entire simulations for four different initial conditions.

### Cooling and equilibration phase

In the cooling phase, the temperature is artificially reduced to 300 K by coupling to a Nosé–Hoover thermostat^30–33^. This controlled cooling process leads to substantial recrystallisation rates in all simulations where a solid diamond core remains intact following the expansion. Recrystallisation occurs because the left-over carbon chain molecules are still in contact with the diamond phase, and due to the high temperature, atoms can cross the enthalpic barrier and return to the diamond phase. Looking at the simulation results after the initial expansion phase in Fig. 2b, it is evident that a significant portion of the diamond is graphitized starting from the surface. Bonds were analysed by the nearest neighbours method with a cut-off distance of 1.8 Å. A graphite-like layer structure can clearly be identified, manifesting in the presence of many $sp-2$ bonded carbon atoms. This results in graphite like layers which arrange parallel to each other. Similar structures were also described theoretically as a result of the annealing of diamond^29^. At the edges of the ND, the graphitic layers are bent to form a closed surface similar to the experimentally observed carbon onion structures^34^.

We compared different cooling rates for the state starting at 3000 K. A 100 times slower cooling rate (2500 ps cooling time) reduced the number of diamond atoms to half, and a 1000 times slower cooling (25,000 ps cooling time) to a fifth. This highlights the importance of absolute values of cooling rates in estimating recrystallisation rates. To keep the simulation time manageable, we report results for a total cooling time of 250 ps for all cases. Although substantial recrystallisation rates can be observed at high enough cooling rates, the number of atoms in the diamond structure is almost constant during the second equilibration phase. Therefore, we expect the released and cooled ND parts to be stable long term. However, the size of the diamond clusters is substantially reduced compared to the initial ND cube. Except in the case of the 2000 K simulation, it consists only of a fraction of the entire diamond cube.

### Off-Hugoniot states

**Figure 3:**
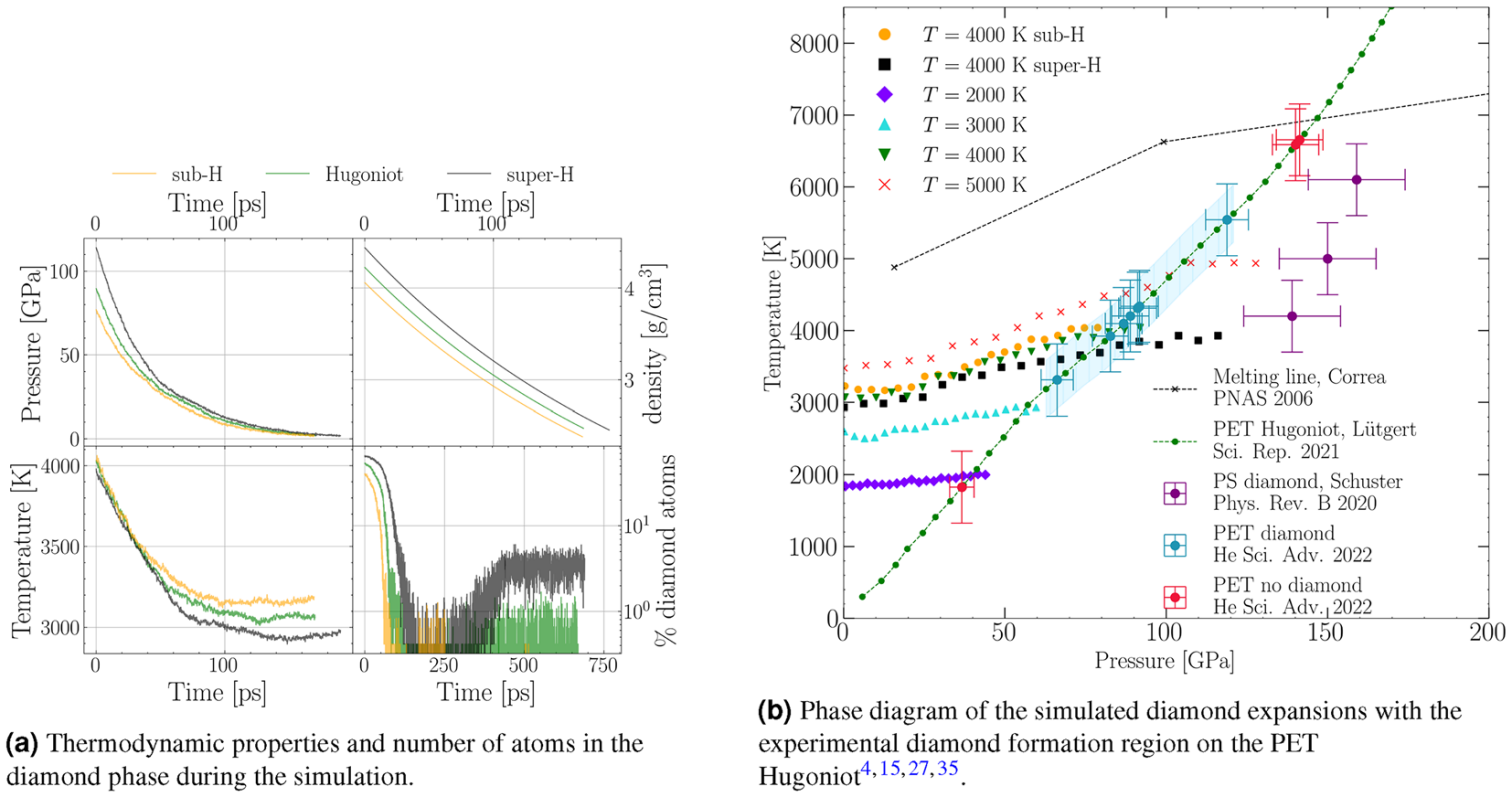
The results of the simulated on- and off-Hugoniot states of the expanding ND cube. **(a)** shows the thermodynamic properties *T*, *P*, and $\rho$ of the off-Hugoniot states compared to the on-Hugoniot states of the same initial pressure. The bottom right graph in **(a)** shows the number of atoms in the diamond phase during the simulations. **(b)** shows the experimentally confirmed diamond nucleation region on the principal PET Hugoniot curve (green line) with the diamond melting line from Ref.^35^. The blue region is where diamonds were found experimentally^15^. The coloured points show all simulated expansions during which *P* and *T* are reduced. The final temperatures are the ones that are reached just before the cooling phase described in detail in Sec. Methods: Molecular Dynamics Simulation. The region where diamond nucleation was observed experimentally from double shock polystyrene (PS) is shown in purple^4^.

For release from 4000 K, two pressure states off the principal Hugoniot of PET were simulated to gauge the effect of pressure on the intact recovery. Off-Hugoniot states were chosen at a lower pressure (sub-H) of 75 GPa and a higher pressure (super-H) of 113 GPa. We use these terms to indicate states that lie below (sub) or above (super) the pressure expected from the Hugoniot curve. The corresponding pressure, density, and temperature curves are displayed along the PET Hugoniot state in Fig. 3a. A comparison to the on-Hugoniot states is shown in Fig. 3b. The pressure drops faster with increased initial pressure, and so does the temperature. The final densities are similar in all simulations, with the low pressure state slightly below the others. However, this simulation had nearly no intact diamond after the first expansion phase, and the final density is very close to ambient graphite. The number of carbon atoms in the diamond phase differ at the start due to the higher surface modifications at lower pressures. This trend is similar during the expansion phase, with the high pressure state leading to more atoms in the diamond lattice. The recrystallisation rates, however, show much larger discrepancies. After the expansion from the super-H off-Hugoniot state, much more atoms return to the diamond phase, while in the sub-H state, no recrystallisation can be observed. Overall, a super-H state, which is experimentally accessible via e.g., two consecutive shock waves is associated with faster release, more diamond after the expansion, and higher recrystallisation rates, while the opposite holds true for the sub-H state.

### Laser shock compression experiment

To experimentally probe the simulated conditions with in situ diagnostics, PET targets were shock compressed with laser intensities from 2.5 to 11 ${{\hbox {TW cm}}^{-2}}$ at two different experiments at the MEC end station at LCLS and at the SACLA facility. Parts of the first dataset have previously been published in Ref.^15^. The pressures ranged from $P=35$ GPa to $P=140$ GPa. The second experiment using a similar configuration was aimed at capturing the release dynamics via later probing times as well as target simplification. The experimental details are found in Section Methods: Laser shock compression.

**Figure 4:**
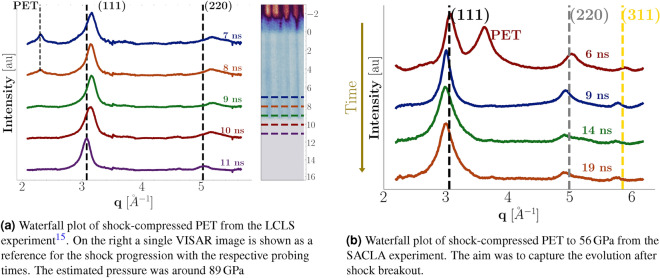
Integrated XRD lineouts at LCLS **(a)** and SACLA **(b)** of shock compressed PET, with different time delays between pump and probe laser. The underlying background has been removed for clarity by applying a polynomial plus a liquid background. The different locations of the PET peak are a result of the orientation of the polymeric chains within the sheets. The peak shift after the breakout is evident.

A waterfall plot of the XRD lineouts from the LCLS data is displayed in Fig. 4a. The axis shows the time delay relative to the arrival time of the drive laser. The dotted lines in the XRD signal are the cold peaks for PET and the (111) and (220) diamond reflections. The shock conditions are estimated from the shock velocity to amount to $T=(4200 \pm 500)$ K and $P = (89 \pm 12)$ GPa at a drive laser intensities of 36 J. The inset to the right shows a VISAR image of a single shot. The laser arrives at $t=0$ which is clear from the reflectivity drop, before which we observe both reflections from the front-side coating and the uncoated rear surface of the target. A small loss of reflectivity is observable before the target is hit, which we attribute to fogging effects from a pre-pulse^36^. The destruction of the targets rear surface leads to the complete loss of reflectivity at breakout time.

A diamond peak forms as soon as 7 ns after laser illumination at an elevated diamond density of $(3.83\pm 0.03)$ ${{\text {g cm}}^{3}}$. The nucleation rates in other hydrocarbon samples at similar conditions were as high as ($>10^{29}$ ${{\hbox {m}}^{-3}\,{\hbox {s}}}$)^4^, and here nucleation occurs already at relatively low pressures in states reached by a single shock Hugoniot compression. As the shock progresses through the sample, the cold PET peak decreases while the diamond peak broadening is reduced. We attribute this effect to the growth of nanocrystallites, and a detailed analysis of the SAXS data led to ND radii around 2 nm^15^. After breakout (9.3 ns), the peaks are shifted to ambient density, indicating a complete release of loading already 1.7 ns after the breakout.

Figure 4b shows the data taken at the SACLA facility, where we chose longer time delays between the pump and probe laser to capture the shock unloading dynamics and make statements about diamond stability after release. The diffraction intensities are plotted as a function of $q=4\pi /(\lambda \sin (\theta ))$ instead of diffraction angle $\theta$ for easier comparability for different X-ray wavelengths $\lambda$. The rear side breakout was about 8.5 ns after illumination, which would place the sample at $(56\pm 3)$ GPa, extending the diamond formation region to lower pressures. The diamond peaks in the compressed sample are shifted to higher angles because of the elevated pressures. The PET peak occurs at higher *q* values due to the different orientation of the PET chains with respect to the probing direction. After the breakout, the peaks are rapidly shifted to lower angles. We attribute this to lattice broadening due to the high temperatures. As the shock breaks out into the vacuum, the pressure drops and the temperature expansion takes effect. In principle, temperature can be inferred from the lattice expansion, assuming a drop to zero pressure via thermal expansion coefficients. We performed Le Bail fits of the lineouts to determine the exact peak positions and, thus, the lattice constants of the diamond. However, the error bars due to laser energy fluctuations were too large to obtain a temperature evolution profile of the diamond peak. Nevertheless, the temperature of the fully released ND was determined to $(3000 \pm 200)$ K from the integration of the thermal expansion coefficient^37^. We assumed the pressure to be fully released. The temperature reduction after breakout is compatible with the cooling of 1000 K predicted from our simulations.

**Figure 5:**
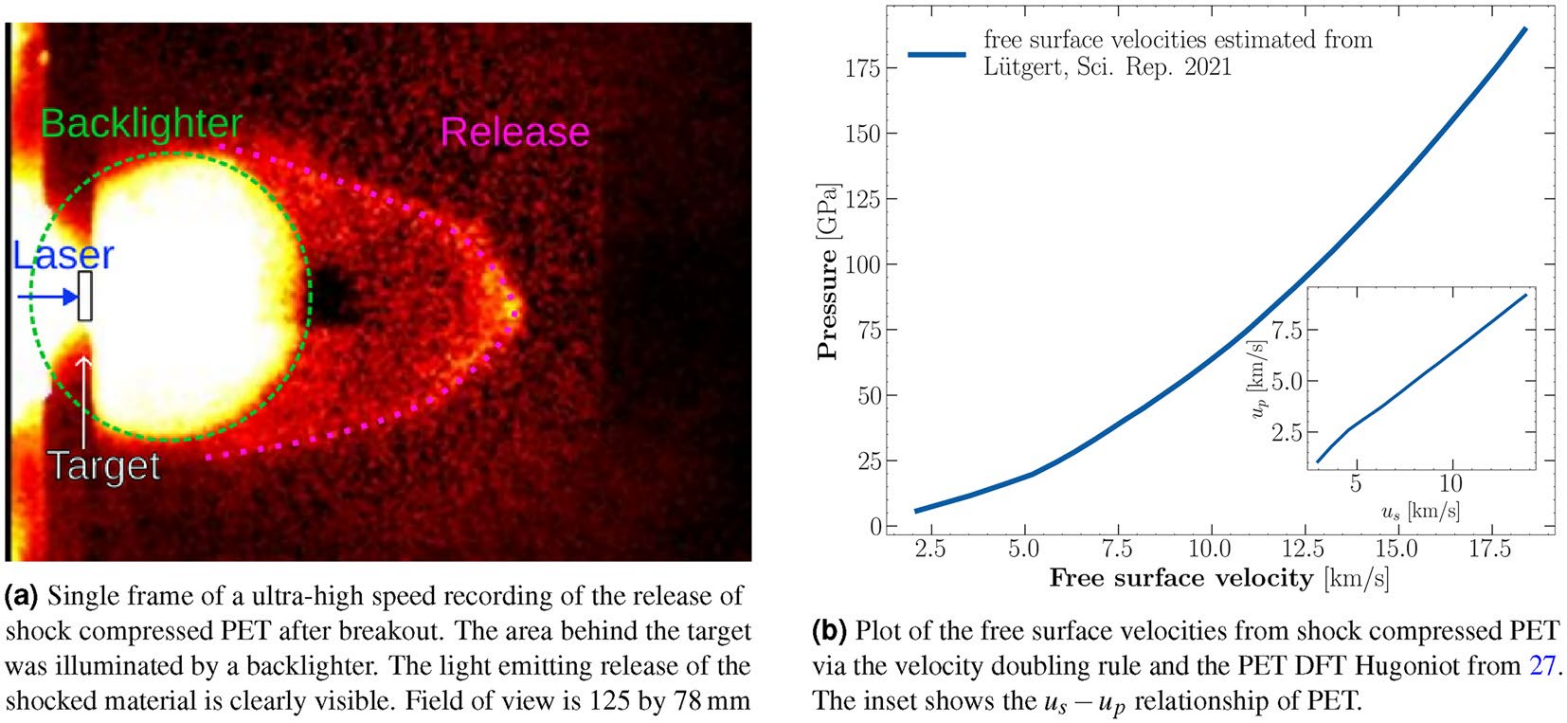
Laser-driven shock compression of PET at the LULI facility. We used the ultra-high speed camera Shimadzu HPV-X2 to image the rear side ejecta after shock breakout and estimate the ejecta velocities via the travelled distance and the inter-frame time. An estimation of of the free surface velocities from previously published Hugoniot data of PET is shown for estimation of ejecta velocities at different pressures.

The velocities of the ejecta front were determined in transversal geometry with a high speed camera. They lay between $(7.0\pm 0.3)$ and ($16.4 \pm 0.2)$ $\hbox {km}/\hbox {s}$. For one shot, a simultaneous measurement of the shock velocity from the VISAR and the high-speed recording was taken. The velocity doubling rule states that the ejecta velocity of a shock breaking out of a free surface into vacuum, is roughly equal to double the particle velocity within that shock^38,39^. It resulted in a free surface velocity of $(12.9 \pm 0.4)$ km/s while the high speed recordings yielded an ejecta velocity of $(13.8\pm 0.3)$ km/s. Therefore, the high-speed camera measurements are very close to the ejecta velocities obtained from the doubling rule. Such comparative measurements are only possible if no catcher is used because the rear side view needs to be unobstructed for VISAR measurements. An example image of those recordings is displayed in Fig. 5a. The typical release shape described in Figure^40^ can be well observed. The good agreement indicates that, in recovery experiments, the transversal high-speed camera setup can give estimates of the shock state. Figure 5b shows the free surface velocity of the rear surface after breakout, estimated from the velocity doubling rule and the PET Hugoniot. This velocity can be a first indicator for intact recovery of NDs in case of subsequent impacts into catcher materials.

## Discussion

In our work, we used MD simulations to study the stability of NDs formed under high-pressure, high-temperature states as they release to ambient conditions. We found the initial Hugoniot conditions to strongly affect ND stability along the release. At the high temperature Hugoniot state around $T = 5000$ K at $P = 122$ GPa, where NDs form in experiments, the number of atoms in the diamond phase drops to zero rapidly, and substantial graphitisation can be observed. In contrast, we observed little change in the numbers of diamond atoms for the 2000 K temperature Hugoniot state. The Hugoniot state at $T=3000$ K and $P=59$ GPa exhibits only a few atoms in the diamond phase after the pressure has released to zero. The on-Hugoniot 4000 K has only 1% of diamond atoms left after the second equilibration. However, a comparison to the off-Hugoniot state for this temperature shows that as the pressure increases, so does the amount of recovered diamond. The final temperature after the expansion decreases as the initial pressure increases. Generalizing, we conclude that lower temperatures are favourable for ND recovery. This is because the main reasons for ND disintegration are graphitisation and bond breaking. The likelihood of both processes increases with temperature. The 2000 K simulation suggests that there exists a limit below which the majority of the diamond remains intact. This scenario can occur if the temperature is sufficiently low, such that the expansion cooling leads to a state within a region where the kinetic barrier of diamond is too high for the system to transform to graphite or liquefy.

Experimentally, we have re-evaluated the previously published data^15^ and amended the dataset with new measurements applying a simplified target design, and extended the formation region down to $(56 \pm 3)$ GPa. Our XRD measurements probed times up to 10.5 ns after shock breakout for the lower pressure states, still showing a clear diamond signal. After breakout, we observed a shift of the diamond peak to ambient density and below, indicating a complete release of pressure already 1.7 ns after breakout, along with temperature-induced lattice broadening. As we do not observe a drop in diamond peak intensity with increased probing delay, even in the highest probed pressure regime, we conclude that most of the diamond remains intact for the timescales investigated. This observation is in clear contrast to the simulated 4000 K state, where almost no diamond remains intact after the expansion. The simulations overestimate the portion of the diamond that is disintegrating substantially upon release. One reason for this might be shielding from the surrounding C–H–O material. Contrary to the simulations, the NDs in the experiment do not expand into vacuum but are surrounded by shocked and releasing material. This might provide some shielding for the ND cores during release and lead to a higher diamond content after expansion. Such behaviour could also be explored theoretically by including the surrounding material explicitly in the simulations, which will require much more understanding of the leftover material mixture and conditions.

Although MD simulations are a valuable and reliable tool, absolute quantities can strongly depend on the choice of interaction potential. In this study, the simulations are used as a tool to deepen our understanding of the release process. We found that the results of our simulations do not match the experimental observations quantitatively but predict the disappearance of diamonds at conditions where they are still visible in the XRD. Despite these quantitative disparities, we argue that the qualitative trends can still be used to find promising approaches, guiding future recovery experiments^16^. The results of the simulations can help guide the design of future experiments, and the absolute values like disintegration and recrystallisation rates should always be benchmarked against experiments. Notably, our experimental study showed a greater presence of diamond post-release compared to simulations, suggesting that simulations should be considered as a conservative estimate or worst-case scenario rather than precise quantitative predictions. Nevertheless, simulations remain invaluable guides for identifying parameter trends and guiding experimental design.

The qualitative trends of the simulations suggest that the sub-Hugoniot releases, experimentally accessible e.g., via double shocks or ramp compression, could be preferable regarding intact recovery of diamond. Less disordering would occur increasing the recrystallisation probability further. We believe that the reason for this is the increased cooling rate at higher initial pressures and overall faster expansion process. It would be interesting to study other metastable high-pressure phases to see if this result is reproducible theoretically. The amount of recovered material could also be compared in single and double shock setups to observe differences in the recovered diamond. Ramp compression allows for even lower temperature states at comparable pressures, but is experimentally more challenging and might be too complex for high repetition rate applications.

Our findings suggest that the lowest temperature where the phase separation and nucleation occur should be chosen to maximise the yields. However, the formation kinetics of NDs at different regimes is not completely understood and both, the nucleation rate and recovery rate need to be taken into account. Furthermore, a deeper understanding of ND nucleation is required across temperature and pressure conditions to find the best combination of nucleation, recrystallisation, and recovery rates.

A notable finding of our simulations is the possibility of recrystallisation after partial disintegration of the diamonds. However, we found those to be dependent on the cooling rates. As we expect them to be even slower in reality than even our longest simulations we are most likely overestimating the recrystallisation if no outside cooling mechanism is provided to the NDs. In line with Ref.^26^ we found no difference in results for cooling times from 100 to 250,ps, but the results differed substantially using longer cooling times. Purely radiative processes would not suffice to provide high enough cooling rates for substantial recrystallisation, although this could occur if a solid diamond core remains intact. Reference^15^ indicates an increase in radius from the size distribution, suggesting growth of the NDs after the breakout. Further investigations into additional sources of cooling or other mechanisms favouring ND growth after breakout is required to understand this discrepancy. Experimentally this can be observed, using techniques like Small Angle X-ray Scattering (SAXS), which can resolve the time evolution of density contrasts on the nanometre scale in shock-compression experiments. Thereby, a possible partial disintegration and growth of solid ND cores during and after shock could be observed using sophisticated models. This can also help with the placement of potential catcher materials in respect to the ejecta or suggest that additional cooling sources might be required in a recovery experiment.

The high speed recordings have determined the ejecta velocities to be in good agreement with the free surface velocities. At the Hugoniot state, where the highest amount of diamond was observed^15^ the release velocity was determined at $(13.8 \pm 0.3)$ $\hbox {km}/\hbox {s}$.

In conclusion, we have carried out MD simulations for the expansion of ND particles from PET on- and off-Hugoniot states into vacuum. The simulations predict a substantial disintegration of the NDs upon expansion, exceeding what is observed experimentally. Recrystallisation is possible but minimal during realistic timescales assuming solely radiative cooling. Higher pressure at similar temperatures can lead to faster cooling during the expansion, and thus less disordering and higher recrystallization rates. Therefore, more recovered material could be expected from double-shocked states. We conducted dynamic compression experiments of PET at similar conditions to the simulations and found diamond XRD signal up to 10.5 ns after breakout. A substantial peak shift to higher angles was observed in states after breakout. We attribute this to thermal broadening and conclude that the pressure has dropped while the temperature is still elevated. High speed recordings of the ejecta enabled us to estimate the impact velocities, which are in good agreement with the free surface velocity obtained from the VISAR. This result encourages high-speed imaging as a possible shock diagnostics in recovery setups with obstructed rear surfaces. Further work should investigate the difference in recovery from single and double-shocked material as well as the impactor-catcher dynamics at the determined velocity.

## Methods

### Molecular dynamics simulation

All calculations described in this work were performed with the environment-dependent interaction potential (EDIP)^41,42^ implemented in LAMMPS^43,44^. EDIPs have been shown to yield accurate descriptions of $sp^3$ fractions at high densities as well as being able to correctly reproduce the stacking of graphitic layers^45^. The first quantity is important in the context of diamond lattice breakdown, while the latter can help describe the predicted formation of carbon onions and in general, graphitisation. The MD timestep was set to 0.1 fs, which was sufficient for energy conservation. Each simulated diamond cube consisted of 4096 atoms and the simulation was split into the following four parts. The NDs are formed behind the shock front in the shocked state and are expected to be in local thermal equilibrium with the shocked material. We study their evolution after the release and their expansion behaviour, taking the already formed NDs at the on- and off-Hugoniot conditions. Modelling the nucleation process itself requires the interaction potentials to accurately represent the breaking of polymeric bonds as well as demixing, which is a very complex process and might require the incorporation of ab initio methods.

First, an initial equilibration of the ND cubes was performed in the NVT ensemble to receive an equilibrium system. The initial equilibration phase was run for 10 ps with fixed boundary conditions. The diamond grid constant and simulation box size were varied to implicitly define the pressure without using a barostat, effectively compressing the unit cells. The *P*-*T* conditions were chosen as the experimentally observed points on the principal Hugoniot where diamond formation was observed^14,15,27,46^.

The expansion and subsequent radiative cooling are assumed to occur on different timescales. The expansion into vacuum is expected to be very fast, while the radiative cooling to take significantly longer. Those hypotheses are supported by the experimental results, which show very fast pressure release and temperature broadening at timings longer after breakout. Therefore, the expansion and cooling phases were separated and simulated independently. However, to reduce computation time to a manageable amount, the cooling phase in vacuum, which can be considered to take orders of magnitude longer than the expansion, was not modelled in its entirety and simply tested in the 3000 K state to understand the general trend.

To simulate the expansion of the diamonds into vacuum we have adjusted the boundary conditions by applying a homogenous dilation at different rates, effectively a slow increase in volume accessible to the system. Contrary to similar studies, the boundary conditions were non-periodic and included reflective walls. This ensures that the entire ND with its surface is modelled. Due to the size of the NDs the release wave traverses a single ND almost instantaneously and we assume isotropic pressure release. Therefore the expansion was applied equally in all directions. Simulations were performed over a wide range of expansion rates chosen similarly to Ref.^26^. The volume was expanded from the shocked equilibrium state to ambient diamond density over a time of 50, 100, 250, 500, 1000 ps and continued until the pressure dropped below zero. The strain rates were kept constant and were defined by the compressed density and the chosen expansion time to reach ambient diamond density. We report these chosen nominal expansion times to ambient diamond density, hoping to give a better intuition than the strain rate.

A qualitative change in the evolution of temperature and diamond stability was observed for expansion times above 500 ps. Above this value, significant diamond disintegration and an increase in temperature were observed. The time until full release in these simulations was between 2 and 4 ns, several times what was observed experimentally, for the entire collection of nanodiamonds in the shocked volume. We therefore excluded these slow-release simulations from the analysis. We are presenting the 50 ps results because the release time to zero pressure was around 200 ps and therefore in better agreement with the experiment. The off-Hugoniot states were simulated using the same expansion rates as the on-Hugoniot state to have comparable results.

The third phase was the cooling phase. Here the cell walls were set as fixed boundaries, and the system was coupled to a Nose-Hoover thermostat. Cooling rates were tested in the 3000 K Hugoniot state, which reduced the final temperature of the system after the expansion (2500 K) to 300 K. For this state, we compared cooling over 250, 2500 and 25, 000 ps, which relates to cooling rates of $10^{13}$, $10^{12}$ and $10^{11}$ K/s. Similar studies did not find a difference in the final state below cooling rates of $10^{14}$ K/s^26^. However, we do find a substantial difference using cooling rates below $10^{13}$ K/s. Purely radiative cooling rates would be even below this value. We are presenting the results for cooling to room temperature over a time span of 250 ps due to limitations in computing time.

The final phase consisted of another equilibration phase for the assessment of long-term stability. The system after cooling was equilibrated in the last obtained state by coupling it again to a Nose–Hoover thermostat at 300 K for 250 ps.

The structural composition of the atoms was analyzed during the equilibration, expansion, and cooling phase with the Common Neighbor Analysis (CNA)^47^ implemented in OVITO^48^. This method gives access to the local embedding of a carbon atom within a diamond lattice via common neighbors. Depending on the second next neighbors of an atom can be either in a perfect diamond lattice, the first or second neighbor of an atom in a diamond lattice, or not related to a diamond lattice at all. This method can be sensitive to thermal fluctuations of the lattice, as interatomic distances can fluctuate substantially at elevated temperatures.

The relative amount of diamond present in each stage can give us an idea of the structural integrity of the diamond lattice along the release path. The integrity of the lattice can give valuable insight into which materials to choose when attempting to recover NDs from laser compression.

### Laser shock compression

The laser compression experiments were performed at the Matter at Extreme Conditions (MEC) end station at the Linear Coherent Light Source (LCLS) and the Spring-8 Angstrom Compact free electron LAser facility (SACLA). The experiment at LCLS was designed to investigate the high-pressure chemistry at planetary interiors, demixing, and possible phase transitions like ND nucleation, while the SACLA experiment was aimed at investigating diamond stability after the breakout at those conditions. At LCLS, this was done by compressing front-side coated (Al) PET targets of 100 $\upmu \hbox {m}$ thickness. The elemental composition of ${\hbox {(C)}_{\text {x}}}$:${({\hbox {H}_{2}}\text {O})_{\text {y}}}$ allows carbon phase separation with leftover water stoichiometry. The targets were compressed with 14–62 J laser pulses delivered over 8 ns on a spot size of 300 $\upmu \hbox {m}$ in diameter. Additional double shock experiments were performed with a 1 : 4 pulse shape with a 7 ns initial shock and 5 ns second shock with pulse energies from 38 to 63 J. At SACLA, the experiment was performed with uncoated PET of 75 $\upmu \hbox {m}$ thickness to see if a simplification of targets is possible. Such a target design would also suppress any unwanted contaminations introduced by aluminum. The laser pulse duration was 5 ns on a spot size of 170 $\upmu \hbox {m}$.

The state along the principal Hugoniot was determined via the transit time method^49,50^. Here the shock velocity $u_s$ is determined by the VISAR breakout timing and the target thickness. In the regime of interest, the relationship between shock and particle velocity can be well approximated by a linear function $u_s=c_0 + \alpha u_p$ compare (Fig. 5b). We used an experimentally benchmarked EOS from DFT/MD simulations^27^ to estimate temperature conditions in the sample, from the measured shock speeds. The measured quantity was the shock speed, which can be translated to pressure and temperature conditions. The uncertainties in temperature represent upper estimates for how accurately we could determine transit times from VISAR in combination with the error bars from the Hugoniot.

Simultaneously XRD measurements were taken at an energy of 9.5 keV at 50 fs pulse duration with an ePix area detector^51^ with $2064\times 1548$ pixels and a pixel size of $50\times 50$ ${\upmu }\hbox {m}$ at LCLS and 10 keV with a flat-panel detector at SACLA. The diffraction data was azimuthally integrated using DIOPTAS^52^.

We performed Le Bail fits of the lineouts to determine the exact peak positions, and thus, lattice constants of the diamond. The fit results for the thermal expansion coefficient of diamond presented in Ref.^37^ have been extrapolated and inverted to relate the peak shape to a temperature. The error bars of the Le Bail fits were very small in comparison, and the analysis was dominated by the fluctuations in the laser energy. Therefore, no clear relation between the time after the breakout and the temperature of the diamond could be inferred.

Additional experiments with the goal of recovering NDs from shock-compressed plastics were carried out at the Laboratoire pour l’utilisation des lasers intenses (LULI) using a Shimadzu HPVX2—ultra-high speed camera HPV-X2 (Shimadzu Corp., Japan). The ultra-high speed camera setup in transversal geometry enabled a comparison of the ejecta velocity from the shock breakout, and the Hugoniot shock state determined via VISAR. The velocity was measured via the inter-frame time and the position of the shock release front. The front point was determined as the pixel value of the release front point at the maximum distance to the target. This velocity can give a first estimate for possible catcher materials. Only the materials in which the NDs remain intact after impact at these high velocities are useful for recovery.

## Data Availability

The datasets generated during the experiments as well as the simulation results are available from the corresponding author on reasonable request.
